# Editorial for the Special Issue on MEMS Technology for Biomedical Imaging Applications

**DOI:** 10.3390/mi10090615

**Published:** 2019-09-16

**Authors:** Qifa Zhou, Yi Zhang

**Affiliations:** 1 Department of Biomedical Engineering and Ophthalmology, University of Southern California, Los Angeles, CA 90007, USA; 2 USC Roski Eye Institute, University of Southern California, Los Angeles, CA 90033, USA

Biomedical imaging is the key technique and process to create informative images of the human body or other organic structures for clinical purposes or medical science. Micro-electro-mechanical systems (MEMS) technology has demonstrated enormous potential in biological imaging applications due to its outstanding advantages of, for instance, miniaturization, high speed, higher resolution, and convenience of batch fabrication. There are many advancements and breakthroughs developing in the academic community, and there are a few challenges raised accordingly upon the designs, structures, fabrication, integration, and applications of MEMS for all kinds of biomedical imaging. This Special Issue of *Micromachines*, entitled “MEMS Technology for Biomedical Imaging Applications”, contains 13 papers (nine articles and four reviews) highlighting recent advances in the field of biomedical imaging and covering broad topics from the key components to the applications of various imaging systems.

In the area of ultrasonic transducers, Brenner et al. reviewed the capacitive micromachined transducers at all levels: Theory and modeling methods, fabrication technologies, system integration, as well as imaging applications [1]. Future trends for capacitive micromachined ultrasonic transducers and their impact within the broad field of biomedical imaging were also discussed. Work by Chen et al. was aimed to provide a piezoelectric array to improve the acoustic field and spatial resolution in medical ultrasonic imaging [2]. Photocurable resin and nano ceramic particles can be 3D-printed into different concentric elements to consist annular piezoelectric arrays, which are capable of tuning the focus zone and lateral resolution. The design, fabrication, and characterization of a tightly focused high frequency needle-type ultrasonic transducer made by Co-doped Na_0.5_Bi_4.5_Ti_4_O_15_ ceramics was demonstrated by Fei et al. [3]. Li et al. also presented tightly focused ultrasonic transducers, which were designed using aluminum nitride thin film as piezoelectric element and using silicon lens for focusing [4]. In addition, a custom designed integrated circuit combining a high frequency wideband low noise amplifier with a common-source and common-gate structure was used to process the ultrasonic medical echo signal with low noise figure, high gain, and good linearity.

This issue has two papers in the field of photoacoustic imaging. Lee et al. reviewed cutting-edge MEMS technologies for photoacoustic imaging and summarizes the recent advances of scanning mirrors and detectors [5]. Conventional silicon and water immersible scanning mirrors were introduced respectively, followed by micromachined transducers, microring resonators, as well as silicon acoustic delay lines and multiplexers. In the work of Qi et al., an optical resolution photoacoustic microscopy system based on a MEMS scanning mirror was proposed [6]. The mirror was used to achieve raster scanning of the excitation optical focus and the photoacoustic signal was detected by a flat transducer in the system.

Two papers on microendoscopy are included in this issue. Qiu et al. presented a review of the advancements of MEMS actuators for optical microendoscopy, including optical coherence tomography, optical resolution photoacoustic microscopy, confocal, multiphoton, and fluorescence wide-field microendoscopy [7]. The work of Yang et al. provided an ultra-thin single-fiber scanner that was electromagnetically driven by a tilted microcoil on a polyimide capillary [8].

This issue also contains three papers in the field of optical microscopy and its key components. Yang et al. reviewed the micro-optical components and their fabrication technologies, focusing on waveguides, mirrors, and microlenses [9]. Further, they emphasized the development of optical systems integrated with these components for in vitro and in vivo bioimaging, respectively. Wang et al. presented an integrated two-dimensional mechanical scanning system using an electrostatic actuator and a SU-8 rib waveguide with a large core cross section [10]. Work by Seo et al. demonstrated an electrostatic MEMS micromirror for high definition and high frame rate Lissajous scanning [11]. The micromirror comprised a low Q-factor inner mirror and frame mirror, which provided two-dimensional scanning at two similar resonant scanning frequencies with high mechanical stability.

Furthermore, Fawole et al. presented two techniques for monitoring the response of smart hydrogels composed of synthetic organic materials that can be engineered to respond (swell or shrink, change conductivity and optical properties) to specific chemicals, biomolecules, or external stimuli [12]. Either the perturbation of microwave field or the current-voltage characteristics of a field-effect transistor was monitored to correlate the response of hydrogel to chemicals. Tian et al. proposed an adaptive absolute ego-motion estimation method using wearable visual-inertial sensors for indoor positioning [13]. They introduced a wearable visual-inertial device to estimate not only the camera ego-motion, but also the 3D motion of the moving object in dynamic environments. This proposed system has much potential to aid the visually impaired and blind people.

We would like to thank all the authors for submitting their papers to this Special Issue. We also thank all the reviewers for dedicating their time and helping to ensure the quality of the submitted papers.
